# Nicotinamide Mononucleotide Alleviates Bile Acid Metabolism and Hormonal Dysregulation in Letrozole-Induced PCOS Mice

**DOI:** 10.3390/biology13121028

**Published:** 2024-12-08

**Authors:** Caifang Ren, Shuang Zhang, Jianyu Ma, Junjie Huang, Pan Huang, Mingzi Qu, Haoyue Zhao, Zhengrong Zhou, Aihua Gong

**Affiliations:** 1 School of Medicine, Jiangsu University, Zhenjiang 212013, China; 2212313103@stmail.ujs.edu.cn (S.Z.); 3221501097@stmail.ujs.edu.cn (J.H.); phuang@ujs.edu.cn (P.H.); 1000005980@ujs.edu.cn (M.Q.); 3211401148@stmail.ujs.edu.cn (H.Z.); zrzhou@ujs.edu.cn (Z.Z.); 2 College of Animal Science and Technology, Nanjing Agricultural University, Nanjing 210095, China; 2017105027@njau.edu.cn; 3 Hematological Disease Institute of Jiangsu University, Affiliated Hospital of Jiangsu University, Jiangsu University, Zhenjiang 212003, China

**Keywords:** PCOS, androgens, bile acids, NMN, metabolomics

## Abstract

Polycystic ovary syndrome (PCOS) involves complex genetic, metabolic, endocrine, and environmental factors. This study explores the effects of nicotinamide mononucleotide (NMN) in a letrozole-induced PCOS mouse model, focusing on metabolic regulation. Letrozole-induced aromatase inhibition elevated androgen and reduced bile acid levels, linking liver dysfunction and gut imbalance to PCOS. Letrozole-treated mice exhibited disrupted estrous cycles, ovarian congestion, and elevated testosterone. NMN intervention alleviated hyperandrogenism, ovarian abnormalities, and bile acid decline but did not fully restore the estrous cycle or improve lipid profiles. Metabolomic analysis showed that NMN partially reversed bile acid and lipid metabolism disturbances. These findings highlight NMN’s protective role in reducing hyperandrogenism and ovarian cyst formation. However, effective PCOS treatment should target liver and gut metabolism, not just ovarian symptoms, to mitigate systemic effects. Bile acid dysregulation may play a key role in PCOS progression and warrants further investigation.

## Simple Summary

Polycystic ovary syndrome (PCOS) is a complex condition influenced by genetic, metabolic, hormonal, and environmental factors. It often leads to high androgen levels, disrupted ovarian function, and metabolic issues. In this study, we investigated the effects of nicotinamide mononucleotide (NMN) on a mouse model of PCOS, which was induced using letrozole, a drug that blocks estrogen production. Letrozole treatment raised androgen levels, reduced bile acid levels, and caused liver dysfunction and ovarian abnormalities. Mice also showed irregular reproductive cycles and hormone imbalances. NMN treatment helped lower androgen levels, improved ovarian health, and partially restored bile acid levels. However, it did not fully normalize the reproductive cycle or lipid metabolism. These findings suggest that effective PCOS treatments should focus not only on improving ovarian health but also on addressing liver and gut metabolism to reduce hormonal imbalances and other health issues.

## 1. Introduction

Polycystic ovary syndrome (PCOS) is one of the most prevalent endocrine–metabolic disorders in women of reproductive age, affecting approximately 10% to 15% of women globally [1,2,3,4]. PCOS typically presents during adolescence and is characterized by clinical or biochemical hyperandrogenism, chronic anovulation, and polycystic ovarian morphology. Early symptoms often include menstrual irregularities, acne, hirsutism, and infertility, frequently accompanied with metabolic dysfunction. As the condition progresses and with advancing age, women with PCOS face a significantly increased risk of long-term complications, including diabetes, hypertension, dyslipidemia, fatty liver disease, hyperlipidemia, and endometrial cancer, all of which severely impair quality of life [5,6,7,8,9,10,11,12].

The pathogenesis of PCOS is multifaceted, involving genetic, metabolic, endocrine, and environmental factors. Among these, metabolic abnormalities and hormonal dysregulation are regarded as primary contributors to pathogenesis. Research has shown that androgen excess in both human and animal models can directly induce PCOS and significantly accelerate the onset of associated complications [9,13,14,15]. Recent studies have shown that artemisinin-derived molecules can inhibit androgen synthesis, significantly alleviating hyperandrogenemia and polycystic ovarian morphology in patients with PCOS, with approximately 63.16% of patients regaining normal menstrual cycles. However, due to the complex and diverse etiology of PCOS, a single therapeutic agent is insufficient to address the multifactorial challenges posed by this condition [11]. Therefore, developing effective treatment strategies that target its diverse underlying causes has become a key focus and challenge in current research.

A recent large-scale study has revealed that androgen levels in the follicular fluid of women with PCOS are significantly elevated, even in the absence of clinical hyperandrogenemia [16]. This observation suggests that intrafollicular androgen accumulation in PCOS may precede the development of systemic hyperandrogenemia. Aromatase, encoded by CYP19A1, serves as the rate-limiting enzyme in the conversion of androgens to estrogens. Impaired aromatase activity is considered a key contributor to androgen accumulation in the follicles of women with PCOS [17,18,19,20,21]. In addition, patients with PCOS who were not stimulated by exogenous gonadotropins exhibited significantly lower levels of sinus follicular estrogen compared to the controls [20]. This further indicates that insufficient CYP19A1 activity may lead to inadequate androgen-to-estrogen conversion, resulting in androgen accumulation in the ovary, which is considered a key pathogenic factor in PCOS. A decline in aromatase activity can be attributed to various factors, including environmental toxins, medications (e.g., aromatase inhibitors), malnutrition, and genetic mutations [22,23,24,25,26]. However, its systemic implications remain poorly understood, and further research is warranted.

This study utilizes letrozole, an aromatase inhibitor, to develop a PCOS mouse model that replicates the pathological state caused by aromatase insufficiency [4,27]. In adolescent females, CYP19A1 is predominantly expressed in the ovaries, and letrozole effectively induces ovarian dysfunction associated with impaired aromatase activity, resulting in PCOS-like pathologies. While previous studies have examined serum biochemical alterations in letrozole-induced PCOS models, their metabolic characteristics remain insufficiently explored [4]. Advances in metabolomics have significantly enhanced our understanding of PCOS-associated metabolic features. Existing studies have identified associations between specific metabolites in the serum and follicular fluid of patients with PCOS and the disease itself [6,28,29]. However, the mechanisms underlying metabolic abnormalities in PCOS, particularly those arising from aromatase insufficiency, are not well understood, and related metabolomic data are limited. Metabolomics, as a comprehensive analytical approach, enables the systematic investigation of metabolic changes under specific pathological conditions. This study seeks to utilize metabolomic analysis to explore the effects of aromatase insufficiency on the metabolic profiles of PCOS mice. These findings are expected to offer novel insights into the mechanisms of metabolic dysfunction in PCOS and contribute to the identification of potential therapeutic targets.

Research has shown that NMN (nicotinamide mononucleotide), a critical precursor of NAD+, plays a pivotal role in regulating cellular energy metabolism, improving insulin resistance, and facilitating DNA repair. In patients with PCOS, NAD+ levels in ovarian granulosa cells are significantly reduced compared to in controls and are associated with inflammation [30]. Supplementation with NMN has been demonstrated to elevate NAD+ levels in the muscle tissues of patients with PCOS, effectively mitigating metabolic abnormalities such as hyperinsulinemia, obesity, and hepatic lipid accumulation [31]. Furthermore, NMN has been shown to have pronounced metabolic benefits in experimental models of aging, PCOS, and obesity [30,32,33,34,35,36]. Despite these findings, the specific effects of NMN on the metabolomics of letrozole-induced PCOS models, particularly in relation to bile acid metabolism and hormonal regulation, remain inadequately understood. This study employs metabolomic analysis to comprehensively examine the metabolic regulatory role of NMN in a letrozole-induced PCOS mouse model. The results aim to clarify the influence of NMN on serum metabolic profiles in PCOS and contribute to the uncovering of the metabolic mechanisms underlying ovarian androgen excess.

## 2. Materials and Methods

### 2.1. Animals

Mice were obtained from the Experimental Animal Center of Jiangsu University and housed in a standard specific-pathogen-free (SPF) environment. The housing conditions were maintained with a 12 h light/dark cycle, with a temperature range of 21–24 °C and humidity levels between 40 and 70%. The mice had ad libitum access to water and a standard diet compliant with the standard GB14924.3-2010 [37] (https://www.chinesestandard.net/PDF/English.aspx/GB14924.3-2010). This animal study received approval from the Animal Ethics Committee of Jiangsu University (Approval No.: UJS-IACUC-AP-2022121401) and was conducted in accordance with the university’s guidelines for the care and use of laboratory animals.

The PCOS mouse model was established using letrozole (Solarbio, Beijing, China), with NMN (BONTAC, Shenzhen, China) added to the drinking water to evaluate its intervention effects [38]. The experimental protocol was as follows: 45 female C57BL/6J mice, aged 4 weeks, were selected. After one week of acclimatization, the mice were randomly assigned to three groups (15 mice per group): the control group, the letrozole group (LET), and the letrozole and NMN group (LETNMN). The LET and LETNMN groups received an oral dose of letrozole at 1 mg/kg per day, dissolved in 0.5% carboxymethyl cellulose (CMC, Solarbio, Beijing, China), for 21 consecutive days. The control group was administered an equal volume of 0.5% CMC solution. The LETNMN group had 1.5 mM NMN added to their drinking water, while the control and LET groups were provided with regular water. Body weight was recorded every four days throughout the feeding period. At the end of the experiment, the mice were fasted for 6 h and anesthetized with isoflurane (RWD LIFE SCIENCE, Shenzhen, China), and blood samples were collected via the retro-orbital sinus. Ovaries and liver tissues were then dissected and fixed in 4% paraformaldehyde (Proteintech Group, Inc, Wuhan, China) for subsequent analysis.

### 2.2. Analysis of Serum Markers

On the day of sample collection, blood samples were allowed to sit at room temperature for one hour before being centrifuged at 3000 rpm for 15 min. The upper serum layer was carefully aspirated and transferred to a new centrifuge tube, and then immediately stored at −80 °C until further analysis. Serum levels of testosterone, estradiol, and NAD+ were quantified using enzyme-linked immunosorbent assays (ELISAs) in accordance with the manufacturer’s instructions (Byabscience Biotechnology, Nanjing, China). Furthermore, total cholesterol, triglyceride, low-density lipoprotein cholesterol, aspartate aminotransferase, alanine aminotransferase, and total bile acid levels were measured using enzymatic assays (Jiancheng Bioengineering Institute, Nanjing, China).

### 2.3. Estrous Cycle Assessment

The assessment of the estrous cycle was conducted following established methodologies from previous studies [39]. Briefly, from days 16 to 21 of the intervention, vaginal cytology was conducted using optical microscopy (Nikon Corporation, Tokyo, Japan) to determine the estrous phase of the mice, thereby evaluating ovarian function. Vaginal smears were collected daily at the same time, were air-dried, and were stained with Trypan Blue (Shanghai Macklin Biochemical Co., Ltd., Shanghai, China) for 1–2 min. The morphological characteristics of vaginal epithelial cells were then examined under a microscope to assess the estrous stage. The criteria for classification were as follows: the diestrus phase was primarily composed of leukocytes; the proestrus phase was characterized by nucleated epithelial cells; the estrus phase consisted of keratinized epithelial cells; and the metestrus phase included a mixture of keratinized epithelial cells, nucleated epithelial cells, and leukocytes.

### 2.4. Histopathological Analysis

Liver and ovarian samples were fixed in 4% paraformaldehyde and then immersed in 70% ethanol, 90% ethanol and 100% ethanol solutions according to the concentration gradient for 1 h each. Following dehydration, the samples were cleared in xylene (Sinopharm Chemical, Shanghai, China) and transferred to embedding molds for embedding in molten paraffin (Proteintech Group, Inc., Wuhan, China). The paraffin blocks were then sectioned into 4–5 μm thick slices, stained with hematoxylin and eosin (Ranjeck Technology, Beijing, China), and mounted for microscopic examination to assess pathological and morphological changes in the tissues.

### 2.5. LC-MS/MS Untargeted Metabolomics

This study utilized LC-MS/MS to unbiasedly detect dynamic changes in small molecular metabolites with a relative molecular weight of less than 1000 Da. Additionally, bioinformatic analyses were conducted to identify differential metabolites and their associated pathways, elucidating their physiological mechanisms. The detailed procedures are outlined below:

Metabolite Extraction: Mouse serum was thawed on ice, and 20 μL of the serum sample was extracted using 120 μL of pre-cooled 50% methanol. The mixture was vortexed for 1 min and then allowed to stand at room temperature for 10 min. The extraction solution was stored at −20 °C overnight, followed by centrifugation at 4000× *g* for 20 min. The supernatant was collected and transferred to a new 96-well plate. Prior to LC-MS/MS analysis, the samples were stored at −80 °C. Additionally, 10 μL of each extraction was reserved to prepare mixed quality control (QC) samples.

LC-MS/MS Analysis: All samples were analyzed using the LC-MS system following predefined protocols. Chromatographic separation was performed on an UltiMate 3000 UPLC system (Thermo Fisher Scientific, Bremen, Germany), and metabolites were detected with a high-resolution tandem mass spectrometer (Q-Exactive, Thermo Scientific). The mass spectrometry data were preprocessed using XCMS software (version 3.4.1). R software (version 4.0), including the XCMS, CAMERA (version 3.4.1), and metaX toolboxes (version 1.4.16), was utilized for data processing, identifying ions based on retention time (RT) and *m*/*z* values. Metabolites were annotated using the HMDB and KEGG databases by matching the precise molecular mass data (*m*/*z*) of the samples with the database entries. A mass difference of less than 10 ppm between the observed and database values was required for metabolite annotation, leading to first-level identification results. Due to the presence of isomers, first-level identification often yielded multiple metabolites corresponding to a single *m*/*z* value. Therefore, an in-house secondary mass spectral library was established for matching with the secondary mass spectrometry data of the sample metabolites, resulting in high-confidence metabolite identification. For the identified IDMS2 metabolites, additional classification annotation from the HMDB and KEGG databases was included and visualized.

Statistical analyses were primarily conducted using R software (version 4.0). A clustering heatmap was generated using the R package pheatmap, a significant difference analysis was performed with the R package metaX, and PLS-DA analysis was conducted using the R package ropls, which computed the VIP values for each variable. Correlation analysis utilized Pearson’s correlation coefficient from the R package cor. Significant differential metabolites were selected based on three criteria: a *p* value < 0.05 from the *t*-test, a fold change > 1.2, and VIP values computed from PLS-DA analysis. A differential enrichment analysis of KEGG pathways was performed using hypergeometric tests, with functional items with a *p* value < 0.05 considered significantly enriched. We acknowledge the assistance of LC Bio Technology Co., Ltd. (Zhejiang, China). in LC-MS/MS detection and bioinformatic analysis.

### 2.6. Statistical Analyses

All experiments were performed at least in triplicate. Data visualization and statistical analyses were conducted using GraphPad Prism 9.0 and SPSS 24.0 software. Results are expressed as mean ± SEM. Differences among the three groups were assessed using one-way analysis of variance (ANOVA) followed by Tukey’s post-hoc test for multiple comparisons. For comparisons between two groups, an unpaired two-tailed Student’s *t*-test was performed. Statistical significance was defined as *p* < 0.05, with significance levels indicated by asterisks (* *p* < 0.05, ** *p* < 0.01, *** *p* < 0.001, **** *p* < 0.0001).

## 3. Results

### 3.1. Effects of Letrozole and NMN on Mouse Body Weight and Ovarian and Liver Phenotypes

**Figure 1:**
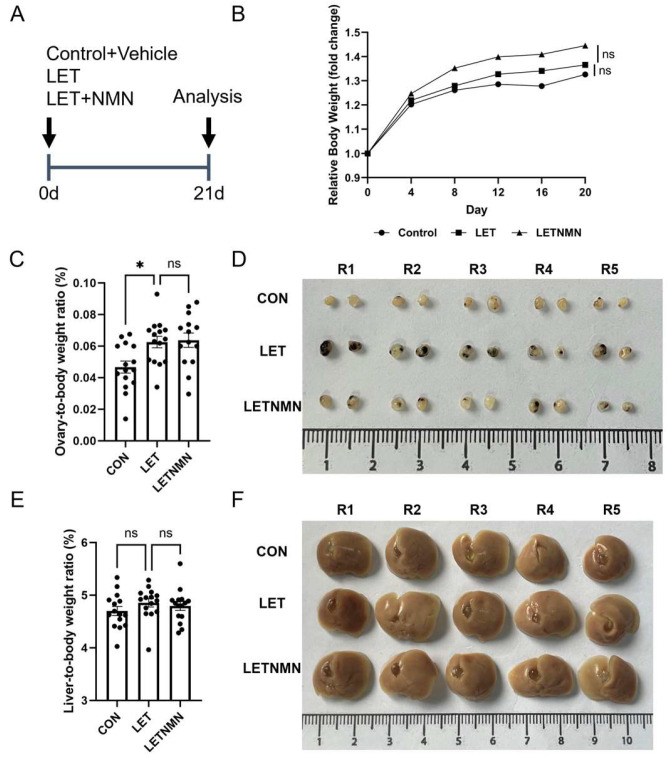
Nicotinamide mononucleotide (NMN) alleviates the polycystic phenotype in letrozole-induced PCOS mice. (**A**) The PCOS model was established in 4-week-old female mice by oral gavage of letrozole (LET, 1 mg/kg/day). The intervention was carried out by administering 1.5 mM NMN via drinking water. At the end of the experiment, the following assessments were performed: (**B**) body weight index (BWI) curve, showing changes relative to initial body weight before gavage; (**C**) ovarian index, calculated as the ratio of ovarian weight to body weight at the time of sampling; (**D**) representative images of ovaries from each group; (**E**) liver index, calculated as the ratio of liver weight to body weight at the time of sampling; (**F**) representative images of livers from each group. Statistical analysis was performed using an unpaired two-tailed Student’s *t*-test: * *p* < 0.05. ns, not significant.

To assess the impact of NMN on the progression of PCOS induced by aromatase inhibition, we established a PCOS model in mice from puberty to adulthood using letrozole. NMN was administered in the drinking water of the intervention group (Figure 1A). No significant differences in body weight were observed among the groups; however, the LET group exhibited a trend toward increased weight compared to the control group (Figure 1B).

Ovarian Phenotype: The ovaries of control mice appeared normal, with only occasional minor red spots noted. In contrast, the ovaries of LET group mice displayed pronounced congestion, deep red coloration, and increased volume. Following NMN intervention, the number of congested spots was reduced, and the trend of ovarian enlargement was alleviated. The ovarian-to-body weight ratio revealed that ovarian weight in LET group mice was significantly higher than that in the control group. However, no significant differences in ovarian index were detected between the LET and LETNMN groups, although some improvement in ovarian congestion was observed in the LETNMN group (Figure 1C,D).

Liver Phenotype: Visual inspection revealed no significant changes in the livers of mice across the groups. Nevertheless, a liver index analysis indicated that the liver index in the LET group was higher than that in the control group, albeit not significantly. The liver index in the LETNMN group did not differ significantly from that in the LET group (Figure 1E,F).

### 3.2. NMN Alleviates Letrozole-Induced Ovarian Cyst Formation and Improves Serum Hormone Homeostasis

**Figure 2:**
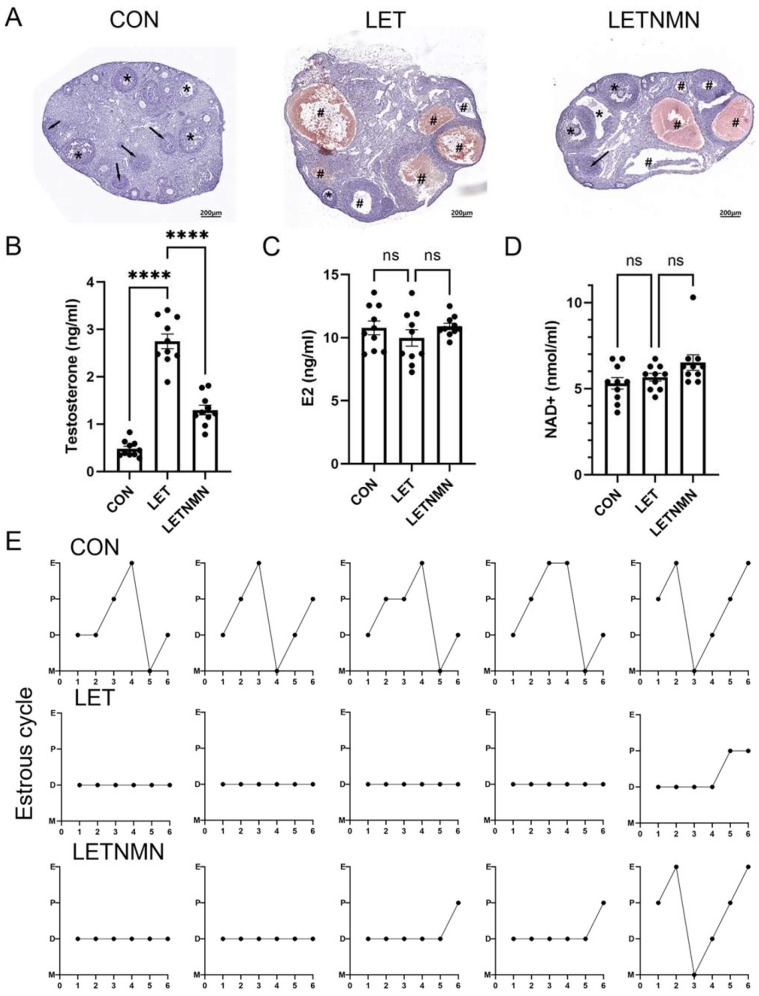
Nicotinamide mononucleotide (NMN) alleviates ovarian morphology abnormalities and hyperandrogenism in letrozole (LET)-induced PCOS mice. (**A**) Representative hematoxylin and eosin (H&E) staining of ovarian tissue, showing cystic follicles (*), antral follicles (#), and corpora lutea (arrows). Scale bar: 200 µm. (**B**) Serum testosterone levels. (**C**) Serum estradiol levels. (**D**) Serum NAD+ levels. (**E**) Estrous cycle analysis: patterns of estrous cycles in five representative mice per group. The *y*-axis abbreviations indicate the following: D—diestrus; E—estrus; M—metestrus; P—proestrus. Hormone levels are presented as mean ± SEM (n ≥ 5 biological replicates). Statistical analysis was performed using an unpaired two-tailed Student’s *t*-test: **** *p* < 0.0001. ns, not significant.

The hematoxylin and eosin (HE) staining of ovarian sections (Figure 2A) indicated an increased number of cystic and antral follicles in the ovaries of LET group mice, accompanied by a decreased count of small follicles and corpora lutea, confirming the successful establishment of a PCOS-like mouse model. While cystic follicles were still present in the LETNMN group, various developmental stages of follicles and corpora lutea were observed.

Serum hormone assays revealed significantly elevated testosterone levels in the LET group compared to the control group. NMN intervention resulted in a notable reduction in testosterone levels, although they remained elevated relative to the control group (Figure 2B). Estradiol levels did not significantly differ among the three groups (Figure 2C). In terms of serum NAD+ levels before and after NMN intervention, NAD+ levels exhibited an increasing trend; however, this change did not reach statistical significance (Figure 2D).

Estrous cycle assessments revealed that 100% of the control group mice exhibited regular estrous cycles. Conversely, LET group mice experienced disrupted estrous cycles, remaining in a prolonged diestrus state. Following NMN intervention, regular estrous cycles were restored in 13% (2/15) of the mice; however, the majority did not recover, indicating that NMN was unable to fully reverse the estrous cycle disruption induced by letrozole (Figure 2E).

### 3.3. NMN Prevents Liver Metabolic Disorders in Letrozole-Induced PCOS Mice

**Figure 3:**
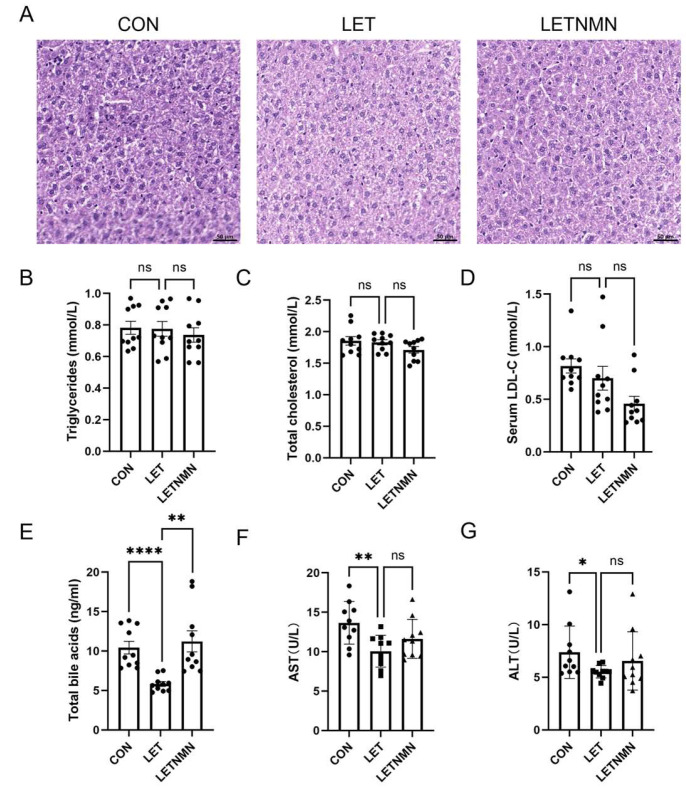
Effects of nicotinamide mononucleotide (NMN) on liver morphology and function in letrozole (LET)-induced PCOS mice. (**A**) Representative hematoxylin and eosin (H&E) staining of liver tissue (scale bar: 200 µm). (**B**) Serum triglyceride levels. (**C**) Serum total cholesterol levels. (**D**) Serum low-density lipoprotein cholesterol (LDL-C) levels. (**E**) Total bile acid levels. (**F**) Aspartate aminotransferase (AST) levels. (**G**) Alanine aminotransferase (ALT) levels. Data are presented as mean ± SEM (n ≥ 5 biological replicates). Statistical analysis was performed using an unpaired two-tailed Student’s *t*-test: * *p* < 0.05, ** *p* < 0.01, **** *p* < 0.0001. ns, not significant.

To evaluate the effects of NMN and letrozole on liver function in PCOS mice, hepatic tissue sections were subjected to hematoxylin and eosin (HE) staining. The results indicated that hepatocytes in the letrozole group displayed pale staining, a condition that was markedly improved after NMN intervention. (Figure 3A). No significant fatty degeneration was detected in any of the groups.

Serum analysis indicated that letrozole had no significant impact on serum triglyceride, total cholesterol, or low-density lipoprotein cholesterol levels, and NMN intervention likewise did not induce notable changes in these parameters (Figure 3B–D). Notably, serum total bile acid levels in the LET group were significantly reduced compared to in the control group, but following 3 weeks of NMN treatment, total bile acid levels were markedly restored (Figure 3E). Furthermore, letrozole significantly reduced alanine aminotransferase (ALT) and aspartate aminotransferase (AST) levels, and NMN intervention partially improved these changes (Figure 3F,G).

### 3.4. Significant Changes in Serum Metabolomics in Letrozole-Induced PCOS Mice

**Figure 4:**
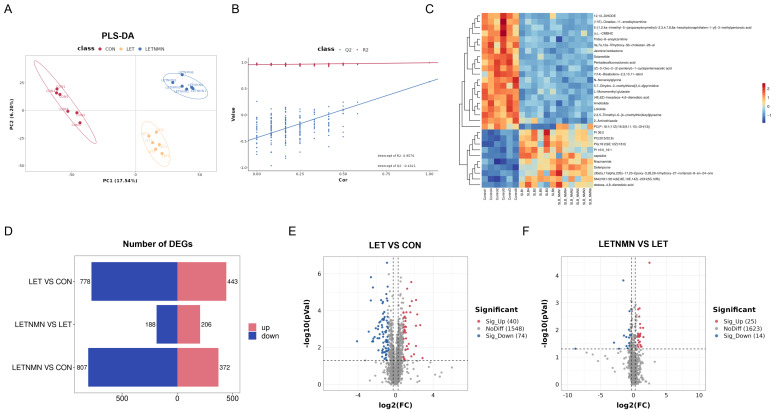
Overview of metabolomic analysis data. (**A**) PLS-DA score plot: The *x*-axis represents the first principal component (PC1), and the *y*-axis represents the second principal component (PC2). Each point corresponds to a sample, with different groups shown in distinct colors. The relative position of the points reflects the degree of variation between samples; the closer the points, the more similar the expression patterns between the samples. (**B**) Permutation test plot: Group labels for each sample are randomly shuffled for modeling and prediction. Each permutation generates a set of R^2^ and Q^2^ values, and regression lines are drawn based on 200 permutations. In the plot, the R² regression line is shown in red, and the Q^2^ regression line in blue. If the R² line stays above the Q^2^ line, and the intercept of the Q^2^ line with the *y*-axis is below 0, the model is considered not overfitted. (**C**) Heatmap of differential metabolites: The heatmap’s *x*-axis represents the samples, and the *y*-axis represents the top 30 differentially expressed metabolites. Different colors indicate relative metabolite abundances: red denotes higher abundance, while blue denotes lower abundance. The deeper the red or blue color, the greater the difference in abundance between groups. (**D**) Differential ion statistics: Based on the quantitative information of ions after data processing, this plot shows the number of differential ions in each comparison group. Red bars indicate significantly upregulated ions, and blue bars indicate significantly downregulated ions. (**E**,**F**) Volcano plot: The overall distribution of differential metabolites in different comparison groups is shown. The *x*-axis represents log2 (FC), and the *y*-axis represents −log10 (*p*-value). Red points indicate significantly upregulated metabolites, blue points indicate significantly downregulated metabolites, and gray points represent metabolites without significant differences. Sig_Up refers to significant upregulation, Sig_Down to significant downregulation, and NoDiff to no significant difference. LET—letrozole; NMN—nicotinamide mononucleotide.

To investigate the impact of letrozole and NMN on metabolite alterations, we conducted non-targeted metabolomic analysis on serum from each group of mice. Partial least squares discriminant analysis (PLS-DA) revealed significant distinctions between the groups, indicating that letrozole and NMN treatments substantially altered serum metabolite composition (Figure 4A,B—PLS-DA score plots and permutation test plots). Clustering heatmap analysis demonstrated clear group-specific clustering, indicating high within-group consistency (Figure 4C). A statistical analysis of differential metabolic ions revealed 443 upregulated and 778 downregulated ions in the LET group compared to the control group, whereas the LETNMN group had 206 upregulated and 188 downregulated ions compared to the LET group (Figure 4D).

A statistical comparison of differential metabolites across groups was conducted based on secondary metabolite annotations, using the criteria of FC ≥ 1.2 or FC ≤ 1/1.2, *p* < 0.05, and VIP > 1. The results are presented in volcano plots; compared to the control group, the LET group exhibited 40 significantly upregulated and 74 significantly downregulated metabolites. In contrast, the LETNMN group showed 25 significantly upregulated and 14 significantly downregulated metabolites compared to the LET group (Figure 4E,F). Detailed lists of differential metabolites for each comparison are provided in Supplementary Tables S1 and S2.

### 3.5. NMN Alleviates Hormonal Metabolism and Abnormalities in Bile Acid Synthesis and Metabolism in Letrozole-Induced PCOS Mice

**Figure 5:**
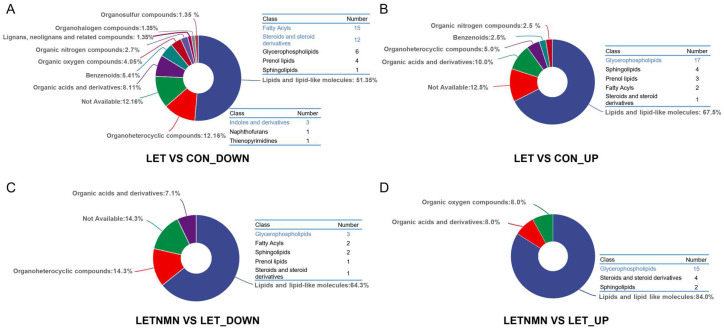
HMDB classification of differential metabolites. The pie charts illustrate the classification of differential metabolites based on the HMDB 4.0 database, while the accompanying tables display the subcategories within each class. (**A**) Classification of downregulated differential metabolites in letrozole (LET) vs. control (CON). (**B**) Classification of upregulated differential metabolites in LET vs. CON. (**C**) Classification of downregulated differential metabolites in LETNMN vs. LET. (**D**) Classification of upregulated differential metabolites in LETNMN vs. LET. NMN—nicotinamide mononucleotide.

Differential metabolites were compared with the HMDB 4.0 database, and a detailed statistical analysis of the metabolites was performed for each group, except for the unannotated metabolites (not available) (Figure 5). Based on the SuperClass classification results in HMDB (shown in the pie chart in Figure 5), the metabolites that were significantly upregulated and downregulated in the LET group compared to the control group were primarily concentrated in three major categories: lipids and lipid-like molecules; organoheterocyclic compounds; and organic acids and derivatives (Figure 5A,B). Furthermore, in the NMN-treated group, the metabolites that were significantly upregulated and downregulated compared to the LET group remained predominantly in the lipids and lipid-like molecules (Figure 5C,D) category, indicating that the regulation of lipid metabolism plays a critical role in both the PCOS model and the NMN intervention process.

From a more detailed HMDB classification perspective (shown in the table in Figure 5), it can be seen that the significantly downregulated metabolites in the LET group compared to the control group included fatty acyls, steroids and steroid derivatives, and glycerophospholipids. Notably, the steroids and steroid derivatives category included bile acid derivatives (Figure 5A). Additionally, indole and its derivatives were the most abundant in the downregulated organoheterocyclic compounds (Figure 5A). In contrast, glycerophospholipids, carboxylic acids and derivatives, and sphingolipids were significantly upregulated in the LET group compared to the control group (Figure 5B). Following NMN intervention, lipid molecules, particularly glycerophospholipids, were significantly upregulated compared to the LET group, while the number of downregulated differential metabolites was relatively low (Figure 5C,D), further confirming the potential role of NMN in improving lipid metabolic disorders.

**Figure 6:**
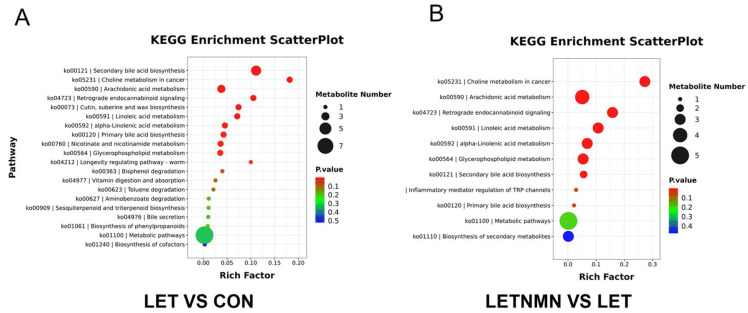
KEGG enrichment analysis of differential metabolites. (**A**) KEGG enrichment plot for differential metabolites in letrozole (LET) vs. control (CON). (**B**) KEGG enrichment plot for differential metabolites in LETNMN vs. LET. Pathway enrichment analysis was conducted using the KEGG pathway database and a hypergeometric test to identify pathways significantly enriched in the differential metabolites compared to the background metabolites identified in this paper. The top 20 pathways with the smallest *p*-values are presented as bubble plots. The RichFactor is defined as the ratio of the number of differential metabolites in a pathway to the total number of metabolites in that pathway. A higher RichFactor indicates a greater degree of enrichment. In the scatterplots, the size of the bubbles reflects the number of differential metabolites in that pathway, and the color represents the *p*-value, indicating the significance of the enrichment. NMN—nicotinamide mononucleotide.

Additionally, differential metabolites were analyzed for enrichment using the KEGG database. While the KEGG database does not encompass all metabolites, we selected the top 20 pathways with the lowest *p*-values for illustration (Figure 6). Compared to the control group, differential metabolites in the LET group were significantly enriched in several metabolic pathways (*p* < 0.01), including secondary bile acid biosynthesis, choline metabolism in cancer, arachidonic acid metabolism, retrograde endocannabinoid signaling, cutin, suberine, and wax biosynthesis, linoleic acid metabolism, alpha-linolenic acid metabolism, primary bile acid biosynthesis, nicotinate and nicotinamide metabolism, and glycerophospholipid metabolism. These results indicate that letrozole profoundly impacted bile acid metabolism pathways in the gut and liver. Following NMN intervention, the KEGG pathways significantly enriched compared to the LET group included choline metabolism in cancer, arachidonic acid metabolism, retrograde endocannabinoid signaling, linoleic acid metabolism, alpha-linolenic acid metabolism, glycerophospholipid metabolism, and secondary bile acid biosynthesis. The enrichment of these pathways suggests that NMN may play a key role in regulating bile acid synthesis and lipid metabolism, potentially mitigating metabolic abnormalities in letrozole-induced PCOS mice. In addition, we selected the top 30 differential metabolites ranked by *p*-value and constructed a network diagram based on these metabolites. This network diagram has been included as Supplementary Material in the revised manuscript to provide a more intuitive visualization of the relationships and potential regulatory interactions among the metabolites (Supplementary Figures S1 and S2).

## 4. Discussion

Hyperandrogenism is one of the key pathogenic factors in polycystic ovary syndrome (PCOS), primarily resulting from excessive androgen synthesis or impaired metabolism. Aromatase, a crucial enzyme responsible for converting androgens to estrogens, plays a pivotal role in this process. Aromatase insufficiency can be triggered by various factors, including environmental toxins, medications (e.g., aromatase inhibitors), malnutrition, and genetic mutations [22,23,24,25,26]. Investigating the role of aromatase dysfunction in PCOS pathogenesis provides valuable insights into its underlying mechanisms.

In this study, we successfully established a PCOS mouse model by inhibiting aromatase with letrozole. The model exhibited hallmark features of PCOS, including follicular cysts, significantly elevated testosterone levels, disrupted estrous cycles, anovulation, and reduced corpus luteum formation, aligning with previous findings on the effects of letrozole on androgen metabolism [40,41,42]. Although letrozole did not significantly decrease serum E2 levels in this study, this finding is consistent with a previous study that found that in pubertal female C57BL/6N mice, five weeks of letrozole treatment led to elevated serum testosterone levels, while E2 levels were maintained within the normal range [43]. This pattern mirrors the hyperandrogenism and follicular-phase estrogen levels observed in women with PCOS [43]. The lack of a significant decrease in E2 may be attributed to compensatory mechanisms or delayed estrogen metabolism and clearance due to impaired hepatic or renal function. In this study, letrozole-treated mice exhibited a significant reduction in granulosa cells within large ovarian follicles, directly leading to decreased estrogen receptor expression. Previous research has demonstrated that female mice with ER-α (Esr1) gene knockout exhibit PCOS-like phenotypes, including polycystic ovaries, anovulation, and corpus luteum deficiency [40,41,44,45,46]. Thus, although serum E2 levels were not significantly reduced, the marked downregulation of ER-α expression in granulosa cells and theca cells suggests disrupted ovarian E2 signaling [41]. Collectively, these findings indicate that PCOS induced by aromatase deficiency is not solely associated with elevated androgen levels but may also involve impaired ovarian E2 signaling and subsequent ovarian-derived androgen accumulation [47,48]. This provides new insights into the role of aromatase deficiency in the pathogenesis of PCOS, highlighting the interplay between androgen excess and disrupted estrogen signaling pathways.

In terms of metabolism, this study found that letrozole treatment did not significantly alter triglyceride or total cholesterol levels, nor did it induce hepatocyte steatosis, consistent with findings from some studies [42]. However, other studies have reported that letrozole treatment significantly increases serum very-low-density lipoprotein (VLDL), low-density lipoprotein (LDL), cholesterol, and triglyceride levels in rats [49]. These discrepancies may stem from differences in experimental models, study designs, and treatment durations. In women with PCOS, the high prevalence of non-alcoholic fatty liver disease (NAFLD) suggests that such metabolic differences might reflect progressive hepatic dysfunction during PCOS development [50]. Moreover, longer experimental durations and sustained androgen elevation may have a more pronounced impact on lipid metabolism, contributing to the variability in findings [42,49]. These results indicate that the metabolic effects of letrozole may vary depending on experimental conditions and individual factors. Future studies should incorporate more systematic and extended observations to comprehensively evaluate letrozole’s impact on metabolic function.

This study found a significant reduction in bile acid levels, including both primary and secondary bile acids, in the serum of letrozole-treated mice, as revealed by total bile acid assays and metabolomic analysis. This contrasts with previous studies on patients with PCOS in which altered bile acid metabolism has been reported. For instance, increased levels of primary and conjugated bile acids in follicular fluid and elevated serum conjugated primary bile acids were positively correlated with hyperandrogenism in patients with PCOS [28,29]. Bile acids and androgens are both derived from cholesterol metabolism, with bile acids retaining a unique steroid structure, making them a critical component in steroid metabolism. Primary bile acids are synthesized in the liver from cholesterol, conjugated with glycine or taurine, and secreted into the intestines, where they are converted to secondary bile acids by gut microbiota [28,51]. In this study, the observed decline in bile acid levels may be attributed to letrozole-induced aromatase inhibition and androgen excess. First, aromatase inhibition may alter cholesterol utilization priorities, diverting cholesterol toward androgen synthesis at the expense of bile acid production. Second, increased hepatic processing of excess androgens may impair liver function, further limiting bile acid synthesis. Additionally, disruptions in liver function and gut microbiota could interfere with bile acid conversion and recirculation. Interestingly, the elevated bile acid levels observed in follicular fluid from patients with PCOS suggest that bile acids may play a critical role in the local follicular microenvironment, potentially regulating steroidogenesis and oxidative stress balance. However, the underlying mechanisms require further investigation. The differences in bile acid alterations between the mouse model and human PCOS may reflect disparities in disease progression and metabolic context, highlighting the limitations of single-factor animal models in fully capturing the complexity of PCOS pathophysiology. Based on the findings of this study and previous data, it can be speculated that abnormalities in bile acid metabolism in the serum of letrozole-induced PCOS mice may result from the combined effects of multiple factors, including endocrine disruption, hepatic–intestinal metabolic dysfunction, gut microbiota imbalance, and chronic inflammation. Future research should focus on the dynamic changes in bile acid metabolism across different models and clinical stages of disease, as well as its impact on the pathophysiology of PCOS. This will help to unravel the metabolic mechanisms underlying PCOS and identify potential therapeutic targets. Although the precise role of bile acids within ovarian follicles remains unclear, several research directions merit further investigation: (1) the metabolic dynamics of bile acids in the ovarian microenvironment of patients with PCOS; (2) the influence of hyperandrogenism on bile acid distribution via endocrine and metabolic pathways; (3) alterations in the expression and function of bile acid transporters; (4) the relationship between bile acids in follicular fluid and ovarian metabolism and inflammatory states; and (5) the effects of bile acid dysregulation on oocyte quality and ovulation rates. These studies could provide critical insights into the link between bile acid metabolism disorders and ovarian dysfunction, such as PCOS, and offer new therapeutic targets for improving reproductive health.

Metabolomic analysis revealed that insufficient aromatase activity led to an excess of ovarian-derived androgens, causing significant alterations in lipid metabolic profiles (Supplementary Table S1). Using annotations from the Human Metabolome Database (HMDB; https://hmdb.ca, accessed on 20 November 2024) and the Kyoto Encyclopedia of Genes and Genomes (KEGG; https://www.genome.jp/kegg/, accessed on 20 November 2024), along with chemical formulas and nomenclature information, we analyzed the primary functions and biological significance of differential metabolites. However, due to the large number of metabolites and the limitations of current metabolite databases, some metabolites could not be annotated to existing pathways or networks. Therefore, we recommend referring to the Supplementary Material Tables for a detailed overview of the various metabolites. The levels of various lipids and their derivatives, particularly fatty acyls and steroid molecules in the serum, were significantly decreased in the letrozole-treated mouse model compared to the control group. Fatty acyl metabolites constituted the largest proportion of downregulated metabolites. For example, (4E,7E,10E)-Hexadeca-4,7,10-trienoylcarnitine is a carnitine ester involved in mitochondrial transport and the β-oxidation of fatty acids; its downregulation may indicate a reduced capacity for fatty acid β-oxidation [52]. Hepoxilin B3, derived from the arachidonic acid metabolic pathway, possesses anti-inflammatory and cytoprotective properties. Its decreased levels may be associated with increased oxidative stress or an imbalance in inflammation regulation [53]. Additionally, 5(S),12(R)-DiHETE (Leukotriene B4, LTB4) is a leukotriene metabolite closely related to inflammation and immune regulation; its downregulation may suggest a weakened immune response or suppressed inflammatory reactions. The downregulation of these metabolites indicates several metabolic abnormalities in the PCOS mouse model: (1) abnormal fatty acid metabolism; (2) oxidative stress and inflammation imbalance; (3) energy metabolism disorders.

Metabolomic analysis also revealed that the administration of letrozole led to the upregulation of metabolites, predominantly among glycerophospholipids, including phosphatidylglycerol (PG), phosphatidylcholine (PC), phosphatidylinositol (PI), phosphatidylserine (PS), and several specific metabolic intermediates (Supplementary Table S1). PG, a crucial component of mitochondrial membranes involved in cellular energy metabolism and membrane stability, was upregulated, possibly reflecting enhanced mitochondrial function or increased membrane synthesis demand—indicating elevated cellular activity or compensatory responses to stress [54,55]. PC, a major constituent of the cell membrane bilayer involved in signal transduction and the regulation of membrane dynamics, showed increased levels, which may reflect enhanced membrane fluidity or adaptive adjustments to oxidative stress [54,55]. PI, a key molecule in signal transduction regulating cell growth, division, and metabolism through pathways such as phosphatidylinositol 3-kinase (PI3K), was upregulated and may be associated with the activation of signaling pathways [56]. PS, essential in apoptotic signaling, membrane flip-flop, and coagulation processes, exhibited increased levels that may indicate enhanced metabolic activity in response to inflammation, immune challenges, or signal transduction stimuli [57,58]. These metabolic alterations suggest that glycerophospholipids play a significant role in the pathogenesis of PCOS, possibly reflecting adaptive cellular responses under stress or metabolic remodeling, providing new insights into the mechanisms of PCOS-related metabolic disorders. Moreover, this study also identified a decrease in tryptophan-derived indole metabolites, which may be linked to gut microbiota dysbiosis, further supporting the hypothesis of gut–liver metabolic dysfunction [59,60]. These findings underscore the intricate interplay between androgen excess, bile acid metabolism, and gut–liver interactions in PCOS, warranting more comprehensive investigations into these pathways.

This study also highlights a novel role of NMN in regulating androgen metabolism via NAD+-dependent pathways and underscores its potential to restore certain ovarian functions. A key finding is that NMN significantly ameliorates hyperandrogenism and provides ovarian protection. NMN may enhance androgen metabolism by increasing 5α-reductase activity and improving mitochondrial function, suggesting its potential utility in preventing PCOS-associated androgen excess [61]. Previous research has shown that during oocyte maturation, levels of NAM in cumulus cells and oocytes increase, accompanied by elevated expression of NAD+-dependent enzymes such as SIRT1 and SIRT4, emphasizing the importance of NAD+ in this process [62,63]. Although NAD+ levels are significantly reduced in the granulosa cells of patients with PCOS, this study observed a slight increase in serum NAD+ levels in letrozole-treated mice. This may be due to compensatory mechanisms in other tissues or a less severe degree of mitochondrial damage. The relatively short experimental duration might have limited the manifestation of chronic metabolic disturbances characteristic of PCOS [30]. Importantly, NMN supplementation significantly enhanced NAD+ availability. Previous studies demonstrated that NMN treatment restored NAD+ levels in the muscle tissue of DHT-induced PCOS mice, effectively alleviating metabolic abnormalities such as hyperinsulinemia, obesity, and hepatic lipid accumulation [31]. The present study further confirms the potential of NMN in mitigating androgen metabolic dysfunction associated with PCOS. These findings not only expand the understanding of NMN’s mechanisms but also provide critical evidence for the development of novel therapeutic strategies for PCOS.

Furthermore, by increasing NAD⁺ availability in letrozole-induced PCOS mice, NMN enhanced bile acid metabolism and downregulated certain metabolites, such as PC (17:0/18:2), PC (18:0/9:0 (CHO)), and PI (17:0_20:3) (Supplementary Table S2). However, it did not fully reverse the damage caused by letrozole. The improvement in bile acid metabolism by NMN may involve several mechanisms: (1) it enhances mitochondrial function and energy metabolism through elevated NAD⁺ levels, thereby restoring the bile acid synthesis capacity of hepatocytes; (2) increased NAD⁺ contributes to improved intestinal barrier function, the regulation of gut microbiota, and the optimization of the enterohepatic circulation of bile acids; (3) the activation of NAD⁺-dependent deacetylases, such as SIRT1, alleviates oxidative stress and inflammation, thus optimizing the microenvironment for bile acid metabolism; and (4) it indirectly promotes the restoration of bile acid levels by ameliorating PCOS-associated metabolic syndrome features, such as lipid metabolism disorders [62,63,64,65,66]. Given that NAD⁺ is a crucial cofactor in numerous enzymatic reactions with extensive biological functions, this study did not confine itself to a specific mechanism but focused on evaluating androgen levels and metabolomic changes directly related to PCOS. The graphical abstract illustrates the effects of NMN on letrozole-induced PCOS mice, particularly its regulatory impact on androgen excess and bile acid metabolic disturbances. Although NMN did not fully reverse the estrous cycle abnormalities or significantly restore altered metabolites in these mice, its potential advantages in metabolic regulation and comprehensive intervention provide new insights for the prevention and treatment of PCOS. These findings offer a significant foundation for further exploration of NMN’s application value and clinical translation in PCOS, warranting in-depth research and validation.

## 5. Conclusions

Letrozole treatment significantly altered the serum metabolomic profiles of mice. Nicotinamide mononucleotide (NMN) effectively alleviated letrozole-induced pathological features in polycystic ovary syndrome (PCOS) mice, including hyperandrogenism, abnormal ovarian morphology, and bile acid metabolic disorders. However, NMN did not fully restore the normal estrous cycle nor reverse the metabolic disturbances caused by letrozole. These findings highlight the complexity of PCOS pathogenesis and emphasize the potential key role of bile acid metabolism in disease progression. We found that NMN enhanced NAD⁺ availability but only partially alleviated metabolic and reproductive abnormalities in letrozole-induced PCOS mice. The role of NAD⁺ in regulating androgen metabolism further underscores NMN’s therapeutic potential in ameliorating specific PCOS symptoms. Our results suggest that NMN, as a potential therapeutic strategy, may alleviate PCOS-related metabolic abnormalities by improving liver–gut axis function and restoring bile acid metabolic balance. These findings provide new evidence supporting NMN’s application in letrozole-induced PCOS mouse models and offer an important reference for future exploration of NMN’s long-term potential in PCOS treatment and its mechanisms in regulating metabolic disorders, particularly bile acid metabolism. The limitations of this study include observations at a single experimental time point and the lack of comprehensive evaluation of dynamic changes in the gut microbiota. Future research will incorporate different disease stages, utilize gut microbiome analyses, and employ targeted lipidomic, bile acid, and steroid metabolomic techniques to more systematically elucidate the interactive mechanisms among the liver, ovary, and intestine in PCOS pathogenesis and treatment.

## Data Availability

Data can be made available through contacting the corresponding author.

## Supplementary Materials

The following supporting information can be downloaded at https://www.mdpi.com/article/10.3390/biology13121028/s1, Table S1: Differential metabolites in LET_VS_CON; Table S2: Differential metabolites in LETNMN_VS_LET. Figure S1: Network in LET_VS_CON; Figure S2: Network in LETNMN_VS_LET.
