# Supplementary figures and images for: Nicotinamide Mononucleotide Alleviates Bile Acid Metabolism and Hormonal Dysregulation in Letrozole-Induced PCOS Mice
Source: Biology (Basel). 2024 Dec 8;13(12):1028. doi: 10.3390/biology13121028 (PMC11673032; doi:10.3390/biology13121028)

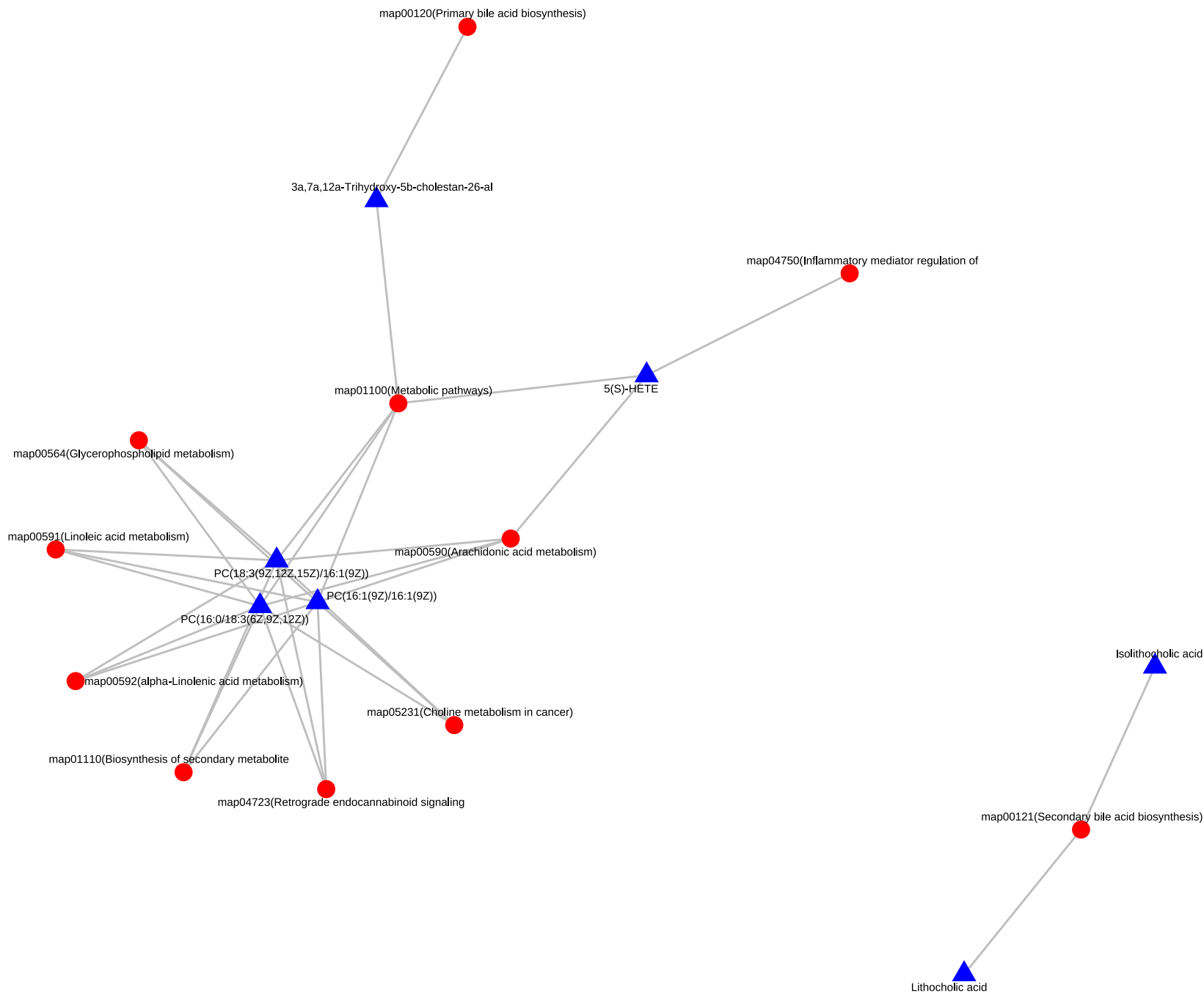

Supplement: Supplementary file 1 [file biology-13-01028-s001.zip › Supplementary Files/Figure S1 LETNMNVSLET_network_top30.pdf]

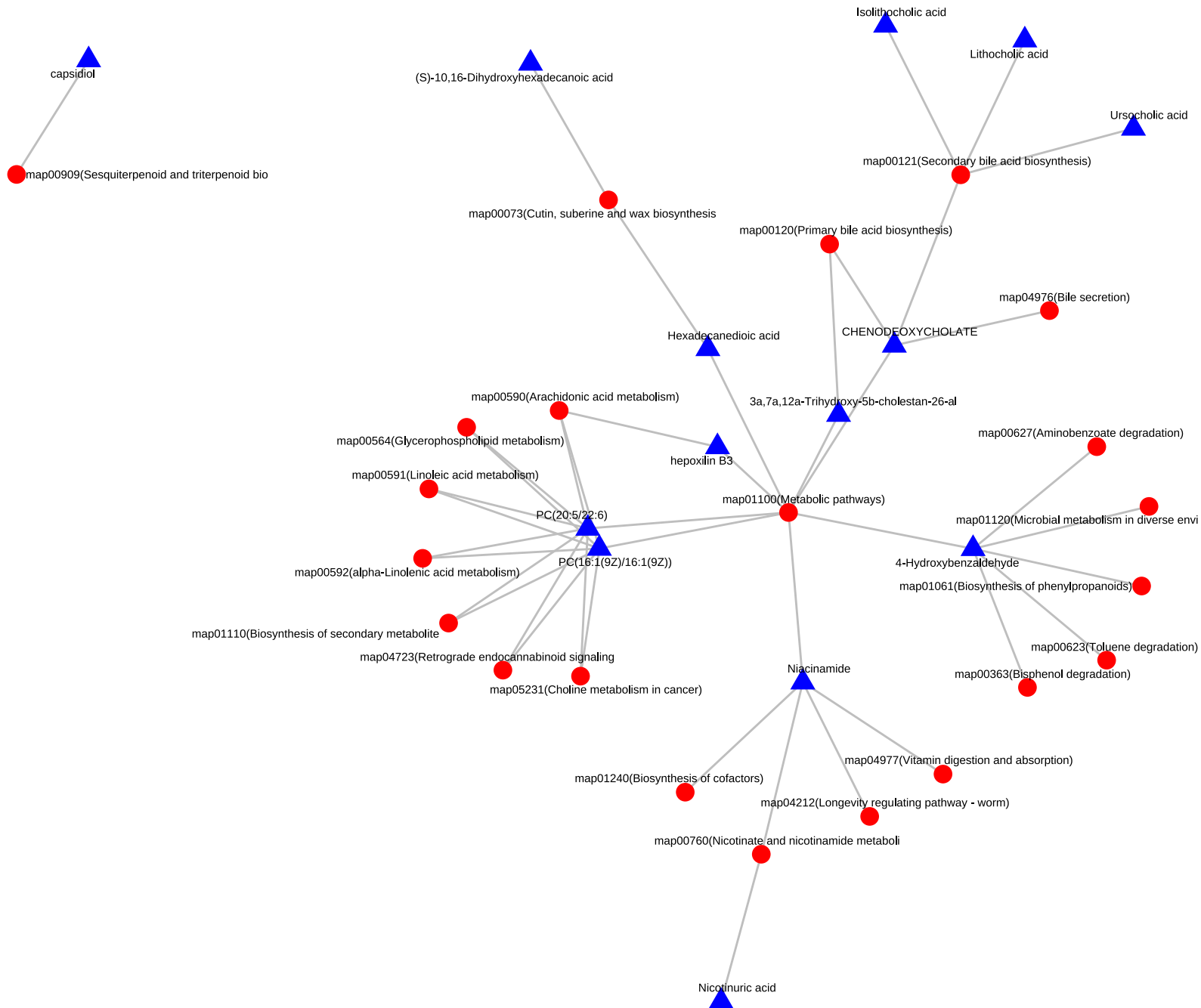

Supplement: Supplementary file 1 [file biology-13-01028-s001.zip › Supplementary Files/Figure S2 LETVSCON_network_top30.pdf]
